# Variation in practice patterns among specialties in the acute management of atrial fibrillation

**DOI:** 10.1186/s12872-015-0009-1

**Published:** 2015-03-12

**Authors:** Ashley M Funk, Keith E Kocher, Jeffrey M Rohde, Brady T West, Thomas C Crawford, James B Froehlich, Sara Saberi

**Affiliations:** Department of Internal Medicine, Division of Cardiovascular Medicine, University of Michigan School of Medicine, Ann Arbor, USA; Department of Emergency Medicine, University of Michigan School of Medicine, 2800 Plymouth Rd., NCRC Bldg 16, Room152S, Ann Arbor, MI 48105-2800 USA; Department of Internal Medicine, Division of General Medicine, University of Michigan School of Medicine, 3119 Taubman Center, 1500 E. Medical Center Dr, Ann Arbor, MI 48109-5376 USA; Survey Research Center, Institute for Social Research, University of Michigan, 426 Thompson Street, Room 4050, Ann Arbor, MI 48104-1248 USA

## Abstract

**Background:**

Atrial fibrillation (AF) is commonly managed by a variety of specialists. Current guidelines differ in their recommendations leading to uncertainty regarding important clinical decisions. We sought to document practice pattern variation among cardiologists, emergency physicians (EP) and hospitalists at a single academic, tertiary-care center.

**Methods:**

A survey was created containing seven clinical scenarios of patients presenting with AF. We analyzed respondent choices regarding rate vs rhythm control, thromboembolic treatment and hospitalization strategies. Finally, we contrasted our findings with a comparable Australasian survey to provide an international reference.

**Results:**

There was a 78% response rate (124 of 158), 37% hospitalists, 31.5% cardiologists, and 31.5% EP. Most respondents chose rate over rhythm control (92.2%; 95% CI, 89.1% - 94.5%) and thromboembolic treatment (67.8%; 95% CI, 63.8% - 71.7%). Compared to both hospitalists and EPs, cardiologists were more likely to choose thromboembolic treatment for new and paroxysmal AF (adjusted OR 2.38; 95% CI, 1.05 - 5.41). They were less likely to favor hospital admission across all types of AF (adjusted OR 0.36; 95% CI, 0.17 - 0.79) but thought cardiology consultation was more important (adjusted OR 1.88, 95% CI, 0.97 - 3.64). Australasian physicians were more aggressive with rhythm control for paroxysmal AF with low CHADS2 score compared to US physicians.

**Conclusions:**

Significant variation exists among specialties in the management of acute AF, likely reflecting a lack of high quality research to direct the provider. Future studies may help to standardize practice leading to decreased rates of hospitalization and overall cost.

**Electronic supplementary material:**

The online version of this article (doi:10.1186/s12872-015-0009-1) contains supplementary material, which is available to authorized users.

## Background

Atrial fibrillation (AF) is the most common arrhythmia encountered in clinical practice, affecting an estimated 5 million adults in the US in 2000 [1] with its prevalence predicted to more than double by 2050 [1,2]. The increasing pervasiveness of AF has been accompanied by increased emergency department (ED) visits [3,4], hospitalizations, and rising healthcare costs. Total national incremental costs for treatment of AF were estimated at US$26 billion in 2008, including US$16.4 billion for hospitalizations (63%) [5].

There are diverse management strategies available for the treatment of AF. Current consensus guidelines established by the American College of Cardiology (ACC), American Heart Association (AHA), American College of Chest Physicians (ACCP), and European Society of Cardiology (ESC) vary in their recommendations and are particularly ambiguous in regards to management in the acute setting [6,7], largely because high quality studies comparing different management strategies are lacking. Variation regarding management decisions such as selecting rate vs rhythm control, timing and need for thromboembolic treatment, as well as cardiology consultation and indications for hospitalization all result in disparate care of patients.

To our knowledge, practice variation among physicians who commonly manage acute presentations of AF has not been evaluated in the US. To address this gap, we designed a survey to (1) document differences in practice patterns in the management of acute presentations of AF between specialties; (2) identify specific areas of management that remain controversial; and (3) contrast these single center US practice patterns with a comparable international study performed in Australia and New Zealand.

## Methods

### Recruitment and setting

All faculty members in the Academic Hospitalist Program, the Division of Cardiovascular Medicine, and Department of Emergency Medicine at a single tertiary academic teaching hospital were invited to participate in an online survey in February 2012 via an email containing a link to the web-based questionnaire. The survey was closed 30 days later. Reminder emails were sent 1 and 3 weeks after the initial invitation and respondents were incentivized with the possibility of winning a nominal reward. The University of Michigan Medical School Institutional Review Board approved this study.

### Survey development and content

The survey contained 7 hypothetical scenarios of patients presenting to the ED with common presentations of acute AF (Table 1). The entire survey is available in Additional file 1: Appendix A. Each case included the patient’s age, presenting symptoms with duration, pertinent past medical history, physical examination findings, CHADS2 score, and the results of relevant diagnostic testing. CHADS2 score was used instead of CHA2DS2-VASc in order to be able to directly compare specific scenarios between the US and Australasian surveys as there was not enough information provided in the Australasian scenarios regarding history of vascular disease to calculate a CHA2DS2-VASc score. Additionally, at the time that this survey was administered, international guidelines still suggested using CHADS2 for stroke risk calculation [6,8,9]. The survey was developed with a 1:1 ratio of scenarios as opposed to attempting to mirror the epidemiology of various presentations of AF in an ED population. This design choice was in line with our objective to describe variation in physician behavior across distinct clinical scenarios; our objective was not to make overall descriptive statements about the target physician population.

**Table 1:** **Clinical scenarios presented in survey**

**Scenario**	**Age**	**Type AF**	**Duration of symptoms**	**CHADS2 Score**	**On warfarin**
**1**	56	New	<48 hours	Low	No
**2**	78	New	< 48 hours	High	No
**3**	50	New	≥ 48 hours	Low	No
**4**	77	New	≥ 48 hours	High	No
**5**	54	Paroxysmal	< 48 hours	Low	No
**6**	76	Paroxysmal	< 48 hours	High	Yes
**7**	82	Chronic	< 48 hours	High	Yes

NOTE: All patients had normal kidney function, no history of structural heart disease, recent negative stress test, no contraindications to anticoagulation, and were not taking any previous cardiac medications.

Low CHADS2 score ≤ 1. *Abbreviation: AF* atrial fibrillation.

The questions following the scenarios explored choice of 1st line therapy aimed at rate or rhythm control and decisions regarding thromboembolic treatment. Anticoagulants include aspirin, warfarin, dabigatran, and rivaroxaban (apixaban was not yet approved for use at the time of survey distribution). Antithrombotics include unfractionated and low-molecular weight heparins. Stand-alone questions explored need for cardiology consultation and hospitalization. The questions were accompanied by fixed possible responses, but most contained an “other” and a free-text option. Information entered in free-text form was coded according to existing categories if possible or “other.” Demographic information including age, gender, years in practice, field of training, and volume of experience was also collected. All answers were non-identifying and respondents had the choice of not providing demographic information.

The survey was developed in collaboration with specialists in survey methodology in the Department of Biostatistics at the School of Public Health and the Institute for Social Research at the University of Michigan. The questionnaire was pre-tested by 9 physicians in training not participating in the study across the same 3 departments and feedback was used to improve the final survey.

### Statistical analysis

Categorical data were summarized using frequencies (%) and their associations were analyzed using chi-square tests. To analyze predictors of choosing a rate control strategy and choosing thromboembolic management, multivariable logistic regression analyses were performed using specialty, type of AF (new diagnosis, paroxysmal, or chronic), CHADS2 score (low = 0 or 1, high ≥ 2), duration of AF (< or ≥ 48 hours), length of time physician has been in practice (≤5 or > 5 years), frequency with which physician encounters acute AF (< once per week or at least once per week), and use of guidelines (ACC, AHA, ESC, American Academy of Family Physicians, or Heart Rhythm Society) as predictor variables. Adjusted odds ratios (OR) with corresponding 95% confidence intervals (95% CI) were calculated for each predictor. Repeated measurements on each physician (across the different scenarios) were accommodated using linearized estimates of standard errors that adjusted for the non-independence of responses introduced by clustering of responses within a physician.

Models of disposition decisions and need for cardiology consultation were also estimated, considering the 3 possible scenarios (new-onset, paroxysmal, and chronic AF). Multivariable logistic regression analyses were performed predicting likelihood to admit and likelihood to consult cardiology adjusting for clustering within any given physician. Predictors in these models included specialty, length of time physician has been in practice, frequency with which physician encounters acute AF, and use of guidelines as above. Adjusted ORs with corresponding 95% CI were calculated.

Finally, we compared practice patterns of these US physicians with those of Australasian physicians documented in a similar survey [10] of EPs and cardiologists. This comparison was planned *a priori* but our survey included newly available oral anticoagulants and did not seek to measure best practice measures. Three scenarios in each survey were identified as equivalent based on type of AF, duration of presenting symptoms, and CHADS2 score. The responses to our questionnaire were recoded to be consistent with the data presented in the Australasian survey and new frequencies were calculated. In the absence of the individual survey responses for the Australasian survey, we viewed the Australasian response distributions as fixed population parameters and performed one-sample chi-square goodness of fit tests for each equivalent pair of scenarios.

Differences were considered statistically significant at p < 0.05. All statistical analyses were performed using the Stata software (Version 13.0).

## Results

There was a 78% (124/158) response rate to the online survey invitation; 31.5% of the respondents were cardiologists, 31.5% were EPs, and 37% were hospitalists. Table 2 depicts the demographic characteristics of the respondents.

**Table 2:** **Demographic characteristics of respondents**

**Characteristic**	**Cardiologists**	**Emergency physicians**	**Hospitalists**	**p-value**
	**n(%)**	**n(%)**	**n(%)**	
**Age**				0.24
**<40**	11 (28)	14 (36)	24 (52)	
**≥40**	18 (46)	17 (44)	10 (22)	
**Missing**	10 (26)	8 (21)	12 (26)	
**Male**	25 (64)	24 (62)	18 (39)	0.033
**Missing**	10 (26)	8 (21)	11 (24)	
**Years of practice**				0.897
**≤5**	11 (28)	13 (33)	17 (37)	
**>5**	19 (49)	18 (46)	18 (39)	
**Missing**	9 (23)	8 (21)	11 (24)	
**AF experience**				0.001
**≥ once per week**	12 (31)	29 (74)	23 (50)	
**< once per week**	18 (46)	3 (8)	12 (26)	
**Missing**	8 (21)	7 (18)	11 (24)	
**Use of guidelines**				0.024
**Yes**	30 (77)	22 (56)	27 (59)	
**No**	0	10 (26)	8 (17)	
**Missing**	9 (23)	7 (18)	11 (24)	

### Rate versus rhythm strategies

**Figure 1:**
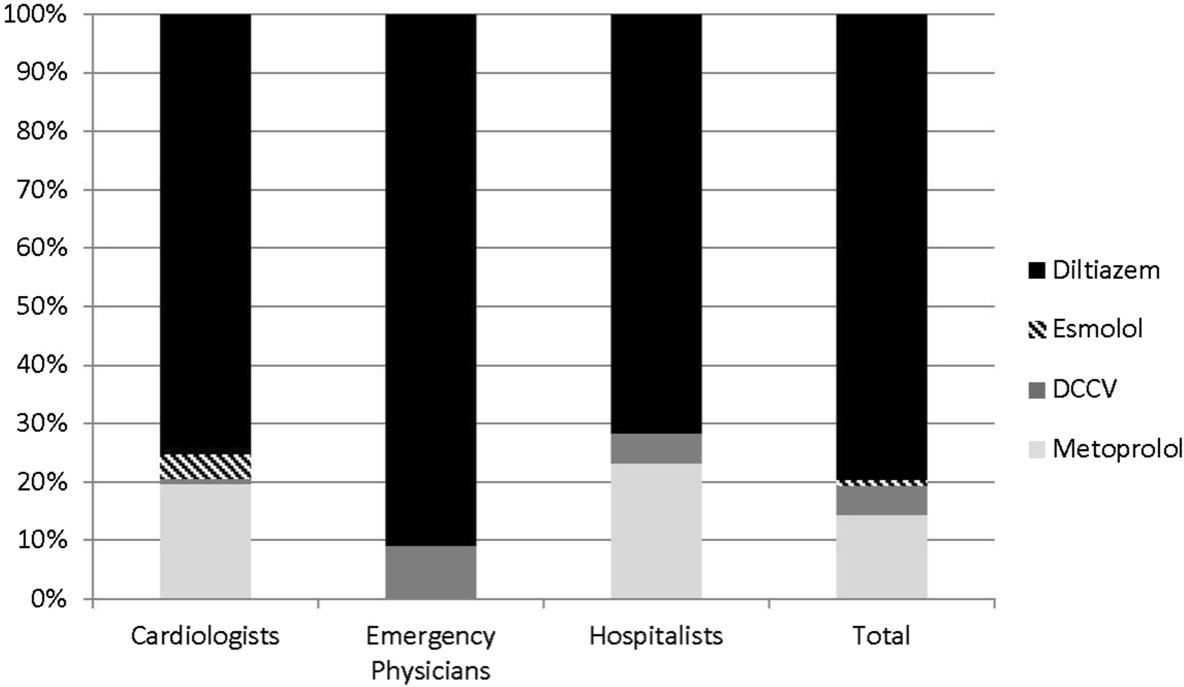
**1st choice management options selected by >1** ***%*** **respondents.** Stacked bar graph depicting the percentage of each specialty that selected Diltiazem, Esmolol, DCCV, or Metoprolol as 1st line management across all scenarios. NOTE: Other choices in the survey not represented in the graph included: Digoxin, Verapamil, Amiodarone, Ibutilide, Procainamide, Propafenone, and Other. There was a significant difference between specialties regarding 1st choice management across all scenarios, p = 0.032. Abbreviation: *DCCV*, direct current cardioversion.

As illustrated in Figure 1, the majority of respondents among all specialties selected rate over rhythm control as their 1st line treatment across all scenarios (92.2%; 95% CI, 89.1% to 94.5%) with diltiazem being the most preferred agent (78%) followed by metoprolol (14%). When a rhythm control strategy was selected, DCCV was most common overall with 21 respondents selecting it as 1st line therapy (5%). Interestingly, DCCV was the only rhythm control strategy employed by EPs and hospitalists, while cardiologists on occasion selected other agents such as amiodarone, ibutilide, or propafenone (all chosen by < 1% of respondents). In multivariable modeling, the adjusted odds of choosing rate control were higher with AF ≥ 48 hours compared to < 48 hours (OR 6.27, 95% CI 2.35 to 16.74) and higher with chronic AF compared with new diagnosis AF (OR 3.09, 95% CI 1.04 to 9.15). Specialty, CHADS2 score, experience with AF, years in practice, and use of current guidelines were not independent predictors of choosing rate control.

### Thromboembolic treatment decisions

**Figure 2:**
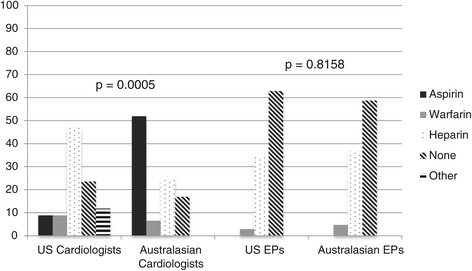
**Thromboembolic treatment.** Bar graph depicting the percentage of each specialty choosing thromboembolic treatment and the percentage that chose bridging in the acute management of AF across scenarios 1–5. There was a significant difference between specialties regarding the decision to use thromboembolic treatment, p = 0.014 as well as the decision to bridge, p < 0.001. NOTE: Abbreviation: *AF*, atrial fibrillation.

Most respondents chose thromboembolic treatment across all scenarios (67.8%; 95% CI 63.8% to 71.7%), though with significant differences among specialties regarding the decision (Figure 2). Cardiologists were more likely to do so in new and paroxysmal AF scenarios than either of the other specialists (OR 2.38, 95% CI 1.05 to 5.41). They were also more likely to choose transesophageal echocardiogram (78.8% vs 69.5% of hospitalists and 57.6% of EPs, p = 0.01) and use antithrombotics (90.4% vs 87.4% of hospitalists and 7.2% of EPs, p < 0.001) prior to cardioversion. In multivariable modeling, the adjusted odds of choosing thromboembolic treatment were higher with a high CHADS2 score (OR 6.89; 95% CI 3.79 to 12.56). A low CHADS2 score and duration of AF < 48 hours predicted a lower odds of bridging (defined as administration of short-acting heparin for antithrombosis until warfarin levels become therapeutic) (OR 0.34, 95% CI 0.17 to 0.68 and OR 0.59, 95% CI 0.39 to 0.91). Most (60%) of those choosing thromboembolic treatment selected a regimen of heparin with subsequent warfarin. EPs used heparin without subsequent long-term anticoagulation the most and avoided newer generation anticoagulants such as dabigatran and rivaroxaban (agents available at the time of the survey distribution) altogether. Hospitalists were more likely than EPs or cardiologists to use warfarin or aspirin alone (36.5%, 9.6%, and 12.9%, respectively). In multivariable modeling, cardiologists and EPs had 4 times higher odds of choosing to bridge than hospitalists (OR 4.02, 95% CI 1.67 to 9.65).

### Admission and consultation decisions

**Figure 3:**
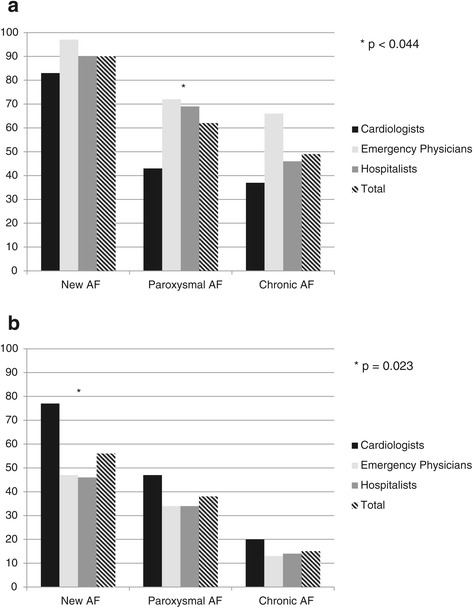
**Likelihood to Admit and Need for Cardiology Consultation** ***.*** **a**: *Likelihood to Admit.* Bar graph comparing percentages of likelihood to admit in scenarios of new vs paroxysmal vs chronic AF. There was a significant difference in admitting practices between specialties for paroxysmal AF (p < 0.044). Abbreviation: *AF*, atrial fibrillation. **b**: *Need for Cardiology Consultation.* Bar graph comparing the percentage of each specialty that thought a cardiology consult would be necessary for scenarios of new vs paroxysmal vs chronic AF. There was a significant difference in need for consultation between specialties for new onset AF (p = 0.023). Abbreviation: *AF*, atrial fibrillation.

The overwhelming majority of respondents in all specialties agreed that scenarios of new presentations of AF (89.7%) required admission (Figure 3a). Cardiologists were least inclined to admit all types of AF (OR 0.36; 95% CI 0.17 to 0.79) and EPs most often favored admission. Specifically, EPs had 15.2 (95% CI 1.26 to 183.73) fold higher odds of admitting cases of new presentations of AF compared to cardiologists. More frequent exposure to AF was associated with a decreased odds of admitting patients in scenarios of new diagnosis AF (OR 0.08; 95% CI 0.01to 0.65) but did not bear any significance on decisions for admitting other types of AF.

All specialists believed cardiology consultation was less important for scenarios of paroxysmal AF (OR 0.46; 95% CI 0.31 to 0.70) and chronic AF (OR 0.12; 95% CI 0.06 to 0.25) compared to new-onset AF (Figure 3b). Despite the fact that cardiologists were least in favor of admitting this patient population, they thought they should be consulted in the management of these patients more so than their colleagues (OR 1.88; 95% CI 0.97 to 3.64).

### International comparison

**Figure 4:**
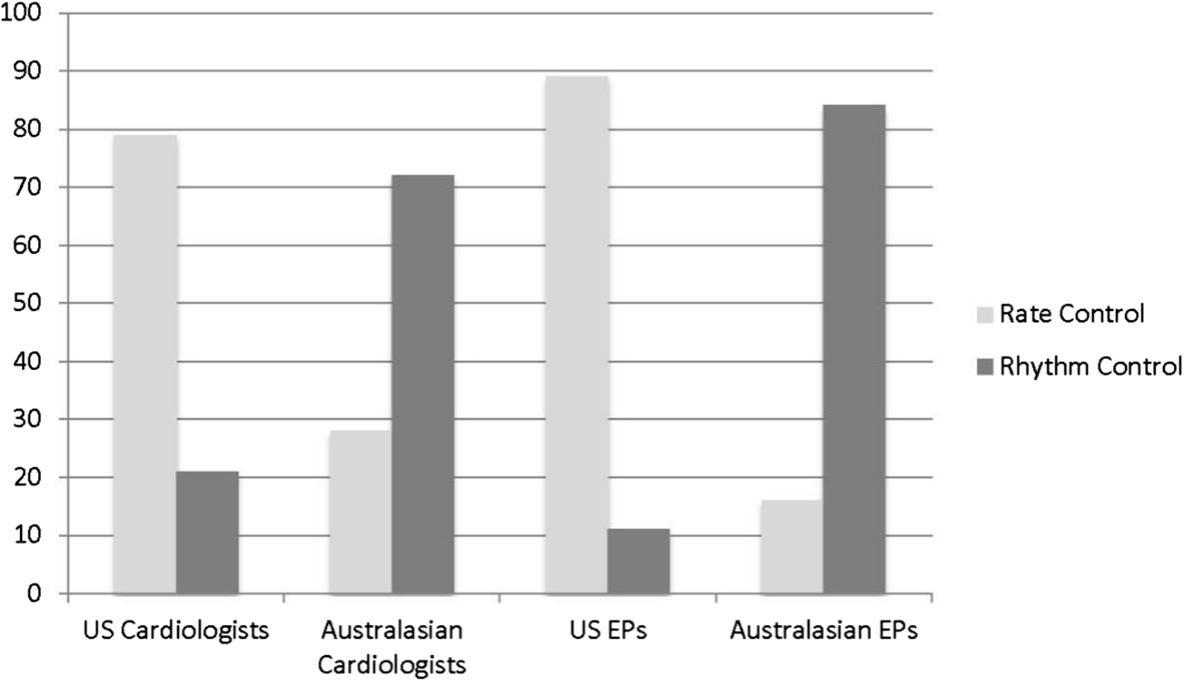
**Australasian** ***versus*** **US Comparison of Rate vs. Rhythm Control Strategies in Paroxysmal AF < 48 hours with Low CHADS2 Score.** Bar graph comparing percentages of rate vs rhythm control as 1st line management for a scenario of paroxysmal AF with symptoms <48 hours and low CHADS2 score. Australasian cardiologists and EPs were more aggressive with rhythm control strategies as compared with US counterparts. Abbreviations: *AF*, atrial fibrillation; *EP*, emergency medicine physicians; *US*, United States.

**Figure 5:**
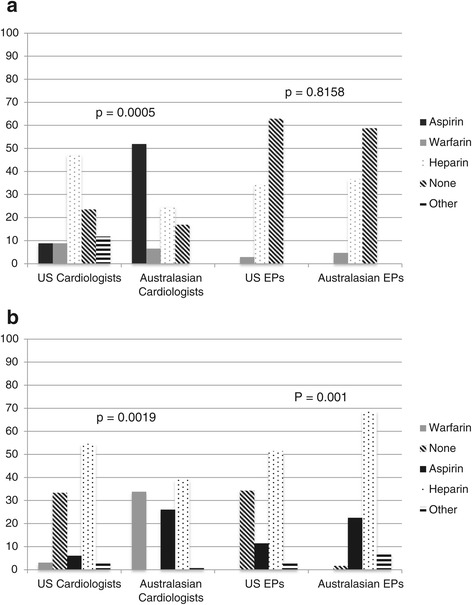
**Australasian versus US comparison in thromboembolic treatment decisions. a**: *Australasian* versus *US Comparison in Thromboembolic Treatment Decisions for Scenarios of Paroxysmal AF < 48 hours with Low CHADS2 Score.* US cardiologists more often chose no thromboembolic treatment, fewer used aspirin, and more selected heparin or other strategies compared to their Australasian counterparts. The category “Other” included Australasian survey responses of clopidogrel, US responses of dabigatran and rivaroxaban, as well as, write-in responses in both surveys. Abbreviations: *AF*, atrial fibrillation; *EP*, emergency medicine physicians; *US*, United States. **b**: *Australasian* versus *US Comparison in Thromboembolic Treatment Decisions for Scenarios of New Onset AF ≥ 48 hours with Low CHADS2 Score.* There were significant differences among both physician groups. US cardiologists chose no thromboembolic treatment and heparin more often, and used aspirin and warfarin alone less often than their Australasian colleagues. US EPs more often selected not to use thromboembolic treatment compared to their Australasian counterparts, and selected aspirin and heparin less frequently. Abbreviations: *AF*, atrial fibrillation; *EP*, emergency medicine physicians; *US*, United States.

The most significant differences in the international comparison were noted in a scenario of paroxysmal AF with symptoms < 48 hours and a low CHADS2 score. Australasian physicians were more aggressive with rhythm control strategies as a 1st line treatment option compared with our physicians (Figure 4) and use of thromboembolic treatment also differed significantly (Figure 5a). Qualitatively, 34% of Australasians chose aspirin while none of their US counterparts did.

For the other two comparable scenarios (both new AF ≥ 48 hours, but one with low and the other with high CHADS2 score), rate control was the preferred 1st line agent by both groups. In the high CHADS2 score scenario, no US physicians chose to anticoagulate with either aspirin or warfarin without bridging, but 26% of Australasian EPs and 8% of cardiologists chose aspirin and 46% of EPs and 84% of cardiologists chose warfarin without bridging. The vast majority of US cardiologists (85%) and EPs (89%) chose heparin therapy. In the low CHADS2 score scenario (Figure 5b), there was significant disagreement regarding thromboembolic treatment. All Australasian cardiologists chose some agent for stroke prevention as opposed to the nearly 1/3rd of their US counterparts who chose none. Qualitatively, 25% of Australiasian EPs chose warfarin alone while none of the US EPs did.

## Discussion

To our knowledge, this is the first study to evaluate practice pattern variation among specialists who frequently encounter acute AF at a single center in the US. The vast majority (92%) of respondents chose rate over rhythm control for acute management of AF. This was in stark contrast to Australasian counterparts who overwhelmingly chose rhythm control strategies. Additionally, there was a high degree of variability among US specialists in the determination of need and type of thromboembolic treatment. Lastly, we observed substantial disagreement among the specialists regarding need for hospital admission and cardiology consultation.

Such international variation in rate vs rhythm control has been documented in other investigations as well. In an international survey of EPs, 94% of US physicians first attempted rate control compared with 70.7% of Canadians, 61.1% of Australasian, and 43.1% of those in the UK [11]. The issue of rate vs rhythm control in the acute management of AF remains unsettled. Several studies suggest a potential benefit of early and aggressive rhythm control in the acute setting [12-17]. A Canadian study of 660 patients by Stiell et al. looked at the efficacy and safety of the Ottawa Aggressive Protocol which treats low risk AF patients with procainamide followed by DCCV if necessary in the ED [18]. They reported a 93.3% conversion rate with 98.6% of patients discharged from the ED. Aggressive ED rhythm management resulted in significant overall hospital savings of tens of thousands of dollars [19], raising the possibility that such strategies may result in fewer hospital admissions and decreased overall cost.

Determination of the timing and need for thromboembolic treatment exhibited the most noteworthy variability in reported practice across physician specialties within our center and internationally, echoing what has been reported by others [20,21]. The confusion is compounded by the vague and sometimes opposing recommendations provided by various guidelines regarding care for those patients with AF duration < 48 hours. The Canadian Cardiovascular Society AF guidelines [9] recommend only aspirin use in conjunction with a rhythm control strategy in those with CHADS2 score of 0, oral anticoagulants for those with CHADS2 scores ≥ 1, and antithrombotic use based on CHADS2 score if cardioversion was successful. The ACCP recommends heparin use prior to elective cardioversion or a TEE-guided approach [6]. The newest ACC/AHA/HRS guidelines [7] recommend that antithrombotics be initiated either before or immediately after cardioversion in those with high risk of stroke (CHA_2_DS_2_-VASc score ≥2) with subsequent long- term anticoagulation. Regarding low-risk patients, they recommend anticoagulation (but no antithrombotics) for the cardioversion itself and no post-cardioversion anticoagulation, a departure from 2006 recommendations [8]. These inconsistencies reflect the fact that no randomized controlled trials comparing thromboembolic treatment strategies in patients with acute AF exist. As decisions regarding thromboembolic treatment become more complex with the introduction of several new oral anticoagulants approved for stroke risk reduction in non-valvular AF, determining the appropriate evidence-based guidance to support provider decision-making in the acute management of AF is critical [22].

### Limitations

This was a single academic-center study; our findings therefore may not be representative of practice patterns at other academic, community or smaller US hospitals but instead reflect our institution’s experience. Also, although scenarios were made to represent common clinical presentations of acute AF with extensive clinical details provided, we cannot be certain that responses reflect real life practice and that all pertinent information clinicians use to make decisions was available for each scenario. Attempts were made to minimize these confounders with the pilot survey. Additionally, there may be an undetected nonresponse bias in our results, given that survey responses were anonymous and we did not have a sampling frame that included demographic information for all physicians. An external population data resource would be needed to determine how closely aligned our survey respondents were with the true population demographics, and to our knowledge, such a resource does not exist for the target physician population of interest. This makes it impossible to know whether the demographic distribution varied between respondents and non-respondents. However, given the high response rate (78%) to our survey, this would seem to be unlikely. Finally, there was a substantial time between our survey and the 2010 Australasian survey, and our survey was not specifically developed for direct comparison to the Australasian survey, although many of the scenario based questions had substantial overlap to allow a basis for evaluation.

## Conclusion

As the prevalence of AF increases and more patients are treated for acute exacerbations in the ED setting, there will be a need for population-level data on the management and outcomes of ED patients with AF in order to improve delivery of care. Based on a survey of cardiologists, EPs, and hospitalists we demonstrated a high degree of practice pattern variation in the management of acute presentations of AF regarding 1st line agents used in rate and rhythm control strategies, decisions regarding thromboemolic treatment, and need for cardiology consultation and hospitalization. The degree of variation noted within our own institution likely reflects a lack of high quality observational and interventional research to help direct the provider. Optimizing the management of acute AF could lead to substantial cost savings if unnecessary testing, interventions, and hospitalizations can be avoided.

## Additional file

Additional file 1:
**Acute Management of Recent-Onset Atrial Fibrillation and Flutter Survey.**

## Abbreviations

AF: Atrial fibrillation
EPs: Emergency physicians
ED: Emergency department
ACC: American college of cardiology
AHA: American heart association
ACCP: American college of chest physicians
ESC: European society of cardiology
DCCV: Direct current cardioversion
TEE: Transesophageal echo
