# Supplementary material for: Variation in practice patterns among specialties in the acute management of atrial fibrillation
Source: BMC Cardiovasc Disord. 2015 Mar 12;15:21. doi: 10.1186/s12872-015-0009-1 (PMC4359491; doi:10.1186/s12872-015-0009-1)
Supplement: Additional file 1: — Acute Management of Recent-Onset Atrial Fibrillation and Flutter Survey. [file 12872_2015_9_MOESM1_ESM.docx]

*Additional file 1: Appendix A*

Survey Title:   Acute Management of Recent-Onset Atrial Fibrillation and Flutter Survey

Thank you for participating in our survey! We really appreciate your time and invite you to share your email address at the end of the survey to be entered into a raffle. Two raffle winners out of approximately 150 respondents will receive an iPAD.

Objective:   The objective of this survey is to determine the current practice patterns among cardiologists, emergency physicians, and hospitalists in the acute management of recent-onset atrial fibrillation and flutter.   This survey is being conducted by a multidisciplinary study team at University of Michigan.   Statement of confidentiality:   Your responses to the following questions will be kept confidential, and the aggregate results, not individual answers, will be published.   Instructions:   This survey will take approximately 20 minutes to complete.   We greatly appreciate your help and commitment to improving our understanding of the management of acute presentations of atrial fibrillation and flutter. Consent: Clicking the "I agree" button below indicates that you have read and understand the above information, and are consenting to participation in this survey.

- I agree
- I do not agree

If I do not agree is selected, skip to end of survey

1. Which of the following best describes the specialty of medicine you practice the majority of your clinical time?

- Cardiology
- Electrophysiology
- Emergency Medicine
- Hospital Medicine
- Other ____________________

SCENARIO 1:  Please read the following scenario and answer the questions based on your general practice pattern.   A 56-year-old man presents to the Emergency Department with 1 day of palpitations and mild shortness of breath.  The patient is clear about the time of symptom onset.  Vital signs: BP 140/75, HR 130s, and SPO2 96%RA.  EKG reveals atrial fibrillation. His CHADS2 Score= 1 (Hypertension).  He does not have a previous history of atrial fibrillation and is not on any anticoagulants or cardiac medications. The patient has normal kidney function, no history of structural heart disease, a recent negative stress test, and no contraindications to anticoagulation.

1. In the acute setting, what would be the first step in your approach to this patient?

- Amiodarone
- Digoxin
- Diltiazem
- Direct Current Cardioversion
- Esmolol
- Ibutilide
- Metoprolol
- Procainamide
- Propafenone
- Verapamil
- Other ____________________

2. If your first choice was unsuccessful, what would be your next choice for management of this patient?

- Amiodarone
- Digoxin
- Diltiazem
- Direct Current Cardioversion
- Esmolol
- Ibutilide
- Metoprolol
- Procainamide
- Propafenone
- Verapamil
- Other ____________________

3. Would you anticoagulate this patient as part of the management of this presentation in the ACUTE setting?

- Yes
- No

If Yes Is Selected go to question #4 [Respondents choosing **a rate control** strategy for question #1 (Digoxin, Diltiazem, Esmolol, Metoprolol, Verapamil) received questions 4a and 4b, respondents choosing a **rhythm control** strategy(Amiodarone, DCCV, Ibutilide, Procainamide, Propafenone) received questions 4c and 4d]. If No is selected, skip to question #5.

4. Which of the following is your first choice for anticoagulation in this ACUTE setting?

- Unfractionated heparin or low molecular weight heparin WITH subsequent warfarin
- Unfractionated heparin or low molecular weight heparin WITHOUT subsequent warfarin
- Dabigatran
- Aspirin
- Warfarin alone
- Other ____________________

4a. If you were to proceed to ELECTRICAL cardioversion for this patient, which of the following would you choose in the ACUTE setting?

- Transesophageal echocardiogram with initiation of heparin or low molecular weight heparin
- Unfractionated heparin or low molecular weight heparin
- Transesophageal echocardiogram without initiation of heparin products
- Warfarin alone
- None of these
- Other ____________________
- Dabigatran

4b. If you were to proceed to CHEMICAL cardioversion for this patient, which of the following would you choose in the ACUTE setting?

- Transesophageal echocardiogram with initiation of heparin or low molecular weight heparin
- Unfractionated heparin or low molecular weight heparin
- Transesophageal echocardiogram without initiation of heparin products
- Warfarin alone
- None of these
- Other ____________________
- Dabigatran

4c. If you were to proceed to ELECTRICAL cardioversion for this patient, would you obtain transesophageal echocardiogram first?

- Yes
- No

4d. If you were to proceed to CHEMICAL cardioversion for this patient, would you obtain transesophageal echocardiogram first?

- Yes
- No

5. If you were to proceed to ELECTRICAL cardioversion for this patient, which of the following would you choose in the ACUTE setting?

- Transesophageal echocardiogram with initiation of heparin or low molecular weight heparin
- Unfractionated heparin or low molecular weight heparin
- Transesophageal echocardiogram without initiation of heparin products
- Warfarin alone
- Dabigatran
- None of these
- Other ____________________

6. If you were to proceed to CHEMICAL cardioversion for this patient, which of the following would you choose in the ACUTE setting?

- Transesophageal echocardiogram with initiation of heparin or low molecular weight heparin
- Unfractionated heparin or low molecular weight heparin
- Transesophageal echocardiogram without initiation of heparin products
- Warfarin alone
- None of these
- Other ____________________
- Dabigatran

7. If the same patient were in atrial flutter NOT atrial fibrillation, would this change your management decisions in the ACUTE setting?

- Yes
- No

If Yes is selected go to question#8. If No is selected, move to Scenario #2.

8. In the acute setting, what would be the first step in your approach to this patient?

- Amiodarone
- Digoxin
- Diltiazem
- Direct Current Cardioversion
- Esmolol
- Ibutilide
- Metoprolol
- Procainamide
- Propafenone
- Verapamil
- Other ____________________

9. If your first choice was unsuccessful, what would be your next choice for management of this patient?

- Amiodarone
- Digoxin
- Diltiazem
- Direct Current Cardioversion
- Esmolol
- Ibutilide
- Metoprolol
- Procainamide
- Propafenone
- Verapamil
- Other ____________________

10. Would you anticoagulate this patient as part of the management of this presentation in the ACUTE setting?

- Yes
- No

If Yes is selected go to question #11 [Respondents choosing **a rate control** strategy for question #8 (Digoxin, Diltiazem, Esmolol, Metoprolol, Verapamil) received questions 11a and 11b, respondents choosing a **rhythm control** strategy for question #8(Amiodarone, DCCV, Ibutilide, Procainamide, Propafenone) received questions 11c and 11d] If No is selected skip to question #12.

11. What is your first choice for anticoagulation in this ACUTE setting?

- Unfractionated heparin or low molecular weight heparin WITH subsequent warfarin
- Unfractionated heparin or low molecular weight heparin WITHOUT subsequent warfarin
- Dabigatran
- Aspirin
- Warfarin alone
- Other ____________________

11a. If you were to proceed to ELECTRICAL cardioversion for this patient, which of the following would you choose in the ACUTE setting?

- Transesophageal echocardiogram with initiation of heparin or low molecular weight heparin
- Unfractionated heparin or low molecular weight heparin
- Transesophageal echocardiogram without initiation of heparin products
- Warfarin alone
- None of these
- Other ____________________
- Dabigatran

11b. If you were to proceed to CHEMICAL cardioversion for this patient, which of the following would you choose in the ACUTE setting?

- Transesophageal echocardiogram with initiation of heparin or low molecular weight heparin
- Unfractionated heparin or low molecular weight heparin
- Transesophageal echocardiogram without initiation of heparin products
- Warfarin alone
- None of these
- Other ____________________
- Dabigatran

11c. If you were to proceed to ELECTRICAL cardioversion for this patient, would you obtain transesophageal echocardiogram first?

- Yes
- No

11d. If you were to proceed to CHEMICAL cardioversion for this patient, would you obtain transesophageal echocardiogram first?

- Yes
- No
- 12. If you were to proceed to CHEMICAL cardioversion for this patient, which of the following would you choose in the ACUTE setting?
- Transesophageal echocardiogram with initiation of heparin or low molecular weight heparin
- Unfractionated heparin or low molecular weight heparin
- Transesophageal echocardiogram without initiation of heparin products
- Warfarin alone
- None of these
- Other ____________________
- Dabigatran

13. If you were to proceed to ELECTRICAL cardioversion for this patient, which of the following would you choose in the ACUTE setting?

- Transesophageal echocardiogram with initiation of heparin or low molecular weight heparin
- Unfractionated heparin or low molecular weight heparin
- Dabigatran
- Transesophageal echocardiogram without initiation of heparin products
- Warfarin alone
- None of these
- Other ____________________

SCENARIO 2: Please read the following scenario and answer the questions based on your general practice pattern. A 78-year-old man presents to the Emergency Department with 5 hours of palpitations and fatigue. Vital signs: BP 130/85, HR 130s, and SPO2 96%RA. EKG reveals atrial fibrillation. He is clear about the time of symptom onset. His CHADS2 Score = 4 (Hypertension, Age, Previous remote ischemic stroke).  He does not have a previous history of atrial fibrillation and is not currently on any anticoagulants or cardiac medications. The patient has normal kidney function, no history of structural heart disease, a recent negative stress test, and no contraindications to anticoagulation.

1. In the acute setting, what would be the first step in your approach to this patient?

- Amiodarone
- Digoxin
- Diltiazem
- Direct Current Cardioversion
- Esmolol
- Ibutilide
- Metoprolol
- Procainamide
- Propafenone
- Verapamil
- Other ____________________

2. If your first choice was unsuccessful, what would be your next choice for management of this patient?

- Amiodarone
- Digoxin
- Diltiazem
- Direct Current Cardioversion
- Esmolol
- Ibutilide
- Metoprolol
- Procainamide
- Propafenone
- Verapamil
- Other ____________________

3. Would you anticoagulate this patient as part of the management of this presentation in the ACUTE setting?

- Yes
- No

If Yes is selected go to question #4 [Respondents choosing **a rate control** strategy for question #1 (Digoxin, Diltiazem, Esmolol, Metoprolol, Verapamil) received questions 4a and 4b, respondents choosing a **rhythm control** strategy for question #1(Amiodarone, DCCV, Ibutilide, Procainamide, Propafenone) received questions 4c and 4d]. If No is selected go to question #5.

4. What is your first choice for anticoagulation in this ACUTE setting?

- Unfractionated heparin or low molecular weight heparin WITH subsequent warfarin (1)
- Unfractionated heparin or low molecular weight heparin WITHOUT subsequent warfarin (2)
- Dabigatran
- Aspirin
- Warfarin alone
- Other ____________________

4a. If you were to proceed to ELECTRICAL cardioversion for this patient, which of the following would you choose in the ACUTE setting?

- Transesophageal echocardiogram with initiation of heparin or low molecular weight heparin
- Unfractionated heparin or low molecular weight heparin
- Transesophageal echocardiogram without initiation of heparin products
- Warfarin alone
- None of these
- Other ____________________
- Dabigatran

4b. If you were to proceed to CHEMICAL cardioversion for this patient, which of the following would you choose in the ACUTE setting?

- Transesophageal echocardiogram with initiation of heparin or low molecular weight heparin
- Unfractionated heparin or low molecular weight heparin
- Transesophageal echocardiogram without initiation of heparin products
- Warfarin alone
- None of these
- Other ____________________
- Dabigatran

4c. If you were to proceed to ELECTRICAL cardioversion for this patient, would you obtain transesophageal echocardiogram first?

- Yes
- No

4d. If you were to proceed to CHEMICAL cardioversion for this patient, would you obtain transesophageal echocardiogram first?

- Yes
- No

5. If you were to proceed to ELECTRICAL cardioversion for this patient, which of the following would you choose in the ACUTE setting?

- Transesophageal echocardiogram with initiation of unfractionated heparin or low molecular weight heparin
- Unfractionated heparin or low molecular weight heparin
- Transesophageal echocardiogram without initiation of heparin products
- Warfarin only
- None of these
- Other ____________________
- Dabigatran

6. If you were to proceed to CHEMICAL cardioversion for this patient, which of the following would you choose in the ACUTE setting?

- Transesophageal echocardiogram with initiation of unfractionated heparin or low molecular weight heparin
- Unfractionated heparin or low molecular weight heparin
- Transesophageal echocardiogram without initiation of heparin products
- Warfarin only
- None of these
- Other ____________________
- Dabigatran

SCENARIO 3: Please read the following scenario and answer the questions based on your general practice pattern.   A 50-year-old woman presents to the Emergency Department with 3 days of worsening fatigue and dyspnea.  Vital signs:  BP 140/75, HR 130s, and SpO2 96%RA. EKG reveals atrial fibrillation.  Her CHADS2 score = 0. She does not have a previous history of atrial fibrillation and is not currently on any anticoagulants or cardiac medications. The patient has normal kidney function, no history of structural heart disease, a recent negative stress test, and no contraindications to anticoagulation.

1. In the acute setting, what would be the first step in your approach to this patient?

- Amiodarone
- Digoxin
- Diltiazem
- Direct Current Cardioversion
- Esmolol
- Ibutilide
- Metoprolol
- Procainamide
- Propafenone
- Verapamil
- Other ____________________

2. If your first choice was unsuccessful, what would be your next choice for management of this patient?

- Amiodarone
- Digoxin
- Diltiazem
- Direct Current Cardioversion
- Esmolol
- Ibutilide
- Metoprolol
- Procainamide
- Propafenone
- Verapamil
- Other ____________________

3. Would you anticoagulate this patient as part of the management of this presentation in the ACUTE setting?

- Yes
- No

If Yes is selected go to question #4 (Respondents choosing **a rate control** strategy for question #1 (Digoxin, Diltiazem, Esmolol, Metoprolol, Verapamil) received questions 4a and 4b, respondents choosing a **rhythm control** strategy for question #1(Amiodarone, DCCV, Ibutilide, Procainamide, Propafenone) received questions 4c and 4d). If No is selected go to question #5.

4. What is your first choice for anticoagulation in this ACUTE setting?

- Unfractionated heparin or low molecular weight heparin WITH subsequent warfarin (1)
- Unfractionated heparin or low molecular weight heparin WITHOUT subsequent warfarin (2)
- Dabigatran
- Aspirin
- Warfarin alone
- Other ____________________

4a. If you were to proceed to ELECTRICAL cardioversion for this patient, which of the following would you choose in the ACUTE setting?

- Transesophageal echocardiogram with initiation of heparin or low molecular weight heparin
- Unfractionated heparin or low molecular weight heparin
- Transesophageal echocardiogram without initiation of heparin products
- Warfarin alone
- None of these
- Other ____________________
- Dabigatran

4b. If you were to proceed to CHEMICAL cardioversion for this patient, which of the following would you choose in the ACUTE setting?

- Transesophageal echocardiogram with initiation of heparin or low molecular weight heparin
- Unfractionated heparin or low molecular weight heparin
- Transesophageal echocardiogram without initiation of heparin products
- Warfarin alone
- None of these
- Other ____________________
- Dabigatran

4c. If you were to proceed to ELECTRICAL cardioversion for this patient, would you obtain transesophageal echocardiogram first?

- Yes
- No

4d. If you were to proceed to CHEMICAL cardioversion for this patient, would you obtain transesophageal echocardiogram first?

- Yes
- No

5. If you were to proceed to CHEMICAL cardioversion for this patient, which of the following would you choose in the ACUTE setting?

- Transesophageal echocardiogram with initiation of unfractionated heparin or low molecular weight heparin
- Unfractionated heparin or low molecular weight heparin
- Transesophageal echocardiogram without initiation of heparin products
- Warfarin only
- None of these
- Other ____________________
- Dabigatran

6. If you were to proceed to ELECTRICAL cardioversion for this patient, which of the following would you choose in the ACUTE setting?

- Transesophageal echocardiogram with initiation of unfractionated heparin or low molecular weight heparin
- Unfractionated heparin or low molecular weight heparin
- Transesophageal echocardiogram without initiation of heparin products
- Warfarin only
- None of these
- Other ____________________
- Dabigatran

SCENARIO 4: Please read the following scenario and answer the questions based on your general practice pattern. A 77-year-old man presents to the Emergency Department with a 4 day history of palpitations and lightheadedness with standing.  Vital signs: BP 130/85, HR 128, SPO2 95%RA. EKG reveals atrial fibrillation. His CHADS2 score = 4 (Age, Diabetes, Previous remote ischemic stroke). He is not on any anticoagulants or cardiac medications. He has no previous history of atrial fibrillation. The patient has normal kidney function, no history of structural heart disease, a recent negative stress test, and no contraindications to anticoagulation.

1. In the acute setting, what would be the first step in your approach to this patient?

- Amiodarone
- Digoxin
- Diltiazem
- Direct Current Cardioversion
- Esmolol
- Ibutilide
- Metoprolol
- Procainamide
- Propafenone
- Verapamil
- Other ____________________

2. If your first choice was unsuccessful, what would be your next choice for management of this patient?

- Amiodarone
- Digoxin
- Diltiazem
- Direct Current Cardioversion
- Esmolol
- Ibutilide
- Metoprolol
- Procainamide
- Propafenone
- Verapamil
- Other ____________________

3. Would you anticoagulate this patient as part of the management of this presentation in the ACUTE setting?

- Yes
- No

If Yes is selected go to question #4 [Respondents choosing **a rate control** strategy for question #1 (Digoxin, Diltiazem, Esmolol, Metoprolol, Verapamil) received questions 4a and 4b, respondents choosing a **rhythm control** strategy for question #1(Amiodarone, DCCV, Ibutilide, Procainamide, Propafenone) received questions 4c and 4d]. If No is selected go to question #5.

4. What is your first choice for anticoagulation in this ACUTE setting?

- Unfractionated heparin or low molecular weight heparin WITH subsequent warfarin (1)
- Unfractionated heparin or low molecular weight heparin WITHOUT subsequent warfarin (2)
- Dabigatran
- Aspirin
- Warfarin alone
- Other ____________________

4a. If you were to proceed to ELECTRICAL cardioversion for this patient, which of the following would you choose in the ACUTE setting?

- Transesophageal echocardiogram with initiation of heparin or low molecular weight heparin
- Unfractionated heparin or low molecular weight heparin
- Transesophageal echocardiogram without initiation of heparin products
- Warfarin alone
- None of these
- Other ____________________
- Dabigatran

4b. If you were to proceed to CHEMICAL cardioversion for this patient, which of the following would you choose in the ACUTE setting?

- Transesophageal echocardiogram with initiation of heparin or low molecular weight heparin
- Unfractionated heparin or low molecular weight heparin
- Transesophageal echocardiogram without initiation of heparin products
- Warfarin alone
- None of these
- Other ____________________
- Dabigatran

4c. If you were to proceed to ELECTRICAL cardioversion for this patient, would you obtain transesophageal echocardiogram first?

- Yes
- No

4d. If you were to proceed to CHEMICAL cardioversion for this patient, would you obtain transesophageal echocardiogram first?

- Yes
- No

5. If you were to proceed to CHEMICAL cardioversion for this patient, which of the following would you choose in the ACUTE setting?

- Transesophageal echocardiogram with initiation of unfractionated heparin or low molecular weight heparin
- Unfractionated heparin or low molecular weight heparin
- Transesophageal echocardiogram without initiation of heparin products
- Warfarin only
- None of these
- Other ____________________
- Dabigatran

6. If you were to proceed to ELECTRICAL cardioversion for this patient, which of the following would you choose in the ACUTE setting?

- Transesophageal echocardiogram with initiation of unfractionated heparin or low molecular weight heparin
- Unfractionated heparin or low molecular weight heparin
- Transesophageal echocardiogram without initiation of heparin products
- Warfarin only
- None of these
- Other ____________________
- Dabigatran

SCENARIO 5:  Please read the following scenario and answer the questions based on your general practice pattern. A 54-year-old woman with a history of PAROXYSMAL atrial fibrillation with 2 prior Emergency Department visits in the past 5 years for atrial fibrillation presents to the ED with a 12 hour history of palpitations. She is clear about the time of symptom onset. Vital signs: BP 140/75, HR 140s, SpO2 96%RA.  EKG reveals atrial fibrillation. Her CHADS2 score = 1 (Hypertension). She is not currently on any anticoagulants or cardiac medications. The patient has normal kidney function, no history of structural heart disease, a recent negative stress test, and no contraindications to anticoagulation.

1. In the acute setting, what would be the first step in your approach to this patient?

- Amiodarone
- Digoxin
- Diltiazem
- Direct Current Cardioversion
- Esmolol
- Ibutilide
- Metoprolol
- Procainamide
- Propafenone
- Verapamil
- Other ____________________

2. If your first choice was unsuccessful, what would be your next choice for management of this patient?

- Amiodarone
- Digoxin
- Diltiazem
- Direct Current Cardioversion
- Esmolol
- Ibutilide
- Metoprolol
- Procainamide
- Propafenone
- Verapamil
- Other ____________________

3. Would you anticoagulate this patient as part of the management of this presentation in the ACUTE setting?

- Yes
- No

If Yes is selected go to question #4 [Respondents choosing **a rate control** strategy for question #1 (Digoxin, Diltiazem, Esmolol, Metoprolol, Verapamil) received questions 4a and 4b, respondents choosing a **rhythm control** strategy for question #1(Amiodarone, DCCV, Ibutilide, Procainamide, Propafenone) received questions 4c and 4d]. If No is selected go to question #5.

4. What is your first choice for anticoagulation in this ACUTE setting?

- Unfractionated heparin or low molecular weight heparin WITH subsequent warfarin (1)
- Unfractionated heparin or low molecular weight heparin WITHOUT subsequent warfarin (2)
- Dabigatran
- Aspirin
- Warfarin alone
- Other ____________________

4a. If you were to proceed to ELECTRICAL cardioversion for this patient, which of the following would you choose in the  ACUTE setting?

- Transesophageal echocardiogram with initiation of heparin or low molecular weight heparin
- Unfractionated heparin or low molecular weight heparin
- Transesophageal echocardiogram without initiation of heparin products
- Warfarin alone
- None of these
- Other ____________________
- Dabigatran

4b. If you were to proceed to CHEMICAL cardioversion for this patient, which of the following would you choose in the  ACUTE setting?

- Transesophageal echocardiogram with initiation of heparin or low molecular weight heparin
- Unfractionated heparin or low molecular weight heparin
- Transesophageal echocardiogram without initiation of heparin products
- Warfarin alone
- None of these
- Other ____________________
- Dabigatran

4c. If you were to proceed to ELECTRICAL cardioversion for this patient, would you obtain transesophageal echocardiogram first?

- Yes
- No

4d. If you were to proceed to CHEMICAL cardioversion for this patient, would you obtain transesophageal echocardiogram first?

- Yes
- No

5. If you were to proceed to CHEMICAL cardioversion for this patient, which of the following would you choose in the ACUTE setting?

- Transesophageal echocardiogram with initiation of unfractionated heparin or low molecular weight heparin
- Unfractionated heparin or low molecular weight heparin
- Transesophageal echocardiogram without initiation of heparin products
- Warfarin only
- None of these
- Other ____________________
- Dabigatran

6. If you were to proceed to ELECTRICAL cardioversion for this patient, which of the following would you choose in the ACUTE setting?

- Transesophageal echocardiogram with initiation of unfractionated heparin or low molecular weight heparin
- Unfractionated heparin or low molecular weight heparin
- Transesophageal echocardiogram without initiation of heparin products
- Warfarin only
- None of these
- Other ____________________
- Dabigatran

7. If the same patient were in atrial flutter NOT atrial fibrillation, would this change your initial management decisions in the ACUTE setting?

- Yes
- No

If Yes is selected go to question #8. If No is selected moves to scenario #6.

8. In the acute setting, what would be the first step in your approach to this patient?

- Amiodarone
- Digoxin
- Diltiazem
- Direct Current Cardioversion
- Esmolol
- Ibutilide
- Metoprolol
- Procainamide
- Propafenone
- Verapamil
- Other ____________________

9. If your first choice was unsuccessful, what would be your next choice for management of this patient?

- Amiodarone
- Digoxin
- Diltiazem
- Direct Current Cardioversion
- Esmolol
- Ibutilide
- Metoprolol
- Procainamide
- Propafenone
- Verapamil
- Other ____________________

10. Would you anticoagulate this patient as part of the management of this presentation in the ACUTE setting?

- Yes
- No

If Yes is selected go to question #11 [Respondents choosing **a rate control** strategy for question #8 [Digoxin, Diltiazem, Esmolol, Metoprolol, Verapamil] received questions 11a and 11b, respondents choosing a **rhythm control** strategy for question #8[Amiodarone, DCCV, Ibutilide, Procainamide, Propafenone] received questions 11c and 11d]. If No is selected, skip to question #12.

11. What is your first choice for anticoagulation in this ACUTE setting?

- Unfractionated heparin or low molecular weight heparin WITH subsequent warfarin
- Unfractionated heparin or low molecular weight heparin WITHOUT subsequent warfarin
- Dabigatran
- Aspirin
- Warfarin alone
- Other ____________________

11a. If you were to proceed to ELECTRICAL cardioversion for this patient, which of the following would you choose in the  ACUTE setting?

- Transesophageal echocardiogram with initiation of heparin or low molecular weight heparin
- Unfractionated heparin or low molecular weight heparin
- Transesophageal echocardiogram without initiation of heparin products
- Warfarin alone
- None of these
- Other ____________________
- Dabigatran

11b. If you were to proceed to CHEMICAL cardioversion for this patient, which of the following would you choose in the  ACUTE setting?

- Transesophageal echocardiogram with initiation of heparin or low molecular weight heparin
- Unfractionated heparin or low molecular weight heparin
- Transesophageal echocardiogram without initiation of heparin products
- Warfarin alone
- None of these
- Other ____________________
- Dabigatran

11c. If you were to proceed to ELECTRICAL cardioversion for this patient, would you obtain transesophageal echocardiogram first?

- Yes
- No

11d. If you were to proceed to CHEMICAL cardioversion for this patient, would you obtain transesophageal echocardiogram first?

- Yes
- No

12. If you were to proceed to CHEMICAL cardioversion for this patient, which of the following would you choose in the ACUTE setting?

- Transesophageal echocardiogram with initiation of heparin or low molecular weight heparin
- Unfractionated heparin or low molecular weight heparin
- Transesophageal echocardiogram without initiation of heparin products
- Warfarin alone
- None of these
- Other ____________________
- Dabigatran

13. If you were to proceed to ELECTRICAL cardioversion for this patient, which of the following would you choose in the ACUTE setting?

- Transesophageal echocardiogram with initiation of heparin or low molecular weight heparin
- Unfractionated heparin or low molecular weight heparin
- Dabigatran
- Transesophageal echocardiogram without initiation of heparin products
- Warfarin alone
- None of these
- Other ____________________

SCENARIO 6: Please read the following scenario and answer the questions based on your general practice pattern. A 76-year-old man with a history of PAROXYSMAL atrial fibrillation on chronic anticoagulation with warfarin presents to the Emergency Department with 24 hours of palpitations.  He is not taking any other cardiac medications. Vital signs: BP 130/85, HR 130s, SpO2 96%RA. EKG reveals atrial fibrillation.  INR 2.5.  His CHADS2 score = 3 (Age, Previous remote ischemic stroke). The patient has normal kidney function, no history of structural heart disease, a recent negative stress test, and no contraindications to anticoagulation.

1. In the acute setting, what would be the first step in your approach to this patient?

- Amiodarone
- Digoxin
- Diltiazem
- Direct Current Cardioversion
- Esmolol
- Ibutilide
- Metoprolol
- Procainamide
- Propafenone
- Verapamil
- Other ____________________

2. If your first choice was unsuccessful, what would be your next choice for management of this patient?

- Amiodarone
- Digoxin
- Diltiazem
- Direct Current Cardioversion
- Esmolol
- Ibutilide
- Metoprolol
- Procainamide
- Propafenone
- Verapamil
- Other ____________________

3. Would you anticoagulate this patient as part of the management of this presentation in the ACUTE setting?

- Yes
- No

If Yes is selected go to question #4 [Respondents choosing **a rate control** strategy for question #1 (Digoxin, Diltiazem, Esmolol, Metoprolol, Verapamil) received questions 4a and 4b, respondents choosing a **rhythm control** strategy for question #1(Amiodarone, DCCV, Ibutilide, Procainamide, Propafenone) received questions 4c and 4d]. If No is selected go to question #5.

4. What is your first choice for anticoagulation in this ACUTE setting?

- Unfractionated heparin or low molecular weight heparin WITH subsequent warfarin (1)
- Unfractionated heparin or low molecular weight heparin WITHOUT subsequent warfarin (2)
- Dabigatran
- Aspirin
- Warfarin alone
- Other ____________________

4a. If you were to proceed to ELECTRICAL cardioversion for this patient, which of the following would you choose in the  ACUTE setting?

- Transesophageal echocardiogram with initiation of heparin or low molecular weight heparin
- Unfractionated heparin or low molecular weight heparin
- Transesophageal echocardiogram without initiation of heparin products
- Warfarin alone
- None of these
- Other ____________________
- Dabigatran

4b. If you were to proceed to CHEMICAL cardioversion for this patient, which of the following would you choose in the  ACUTE setting?

- Transesophageal echocardiogram with initiation of heparin or low molecular weight heparin
- Unfractionated heparin or low molecular weight heparin
- Transesophageal echocardiogram without initiation of heparin products
- Warfarin alone
- None of these
- Other ____________________
- Dabigatran

4c. If you were to proceed to ELECTRICAL cardioversion for this patient, would you obtain transesophageal echocardiogram first?

- Yes
- No

4d. If you were to proceed to CHEMICAL cardioversion for this patient, would you obtain transesophageal echocardiogram first?

- Yes
- No

5. If you were to proceed to CHEMICAL cardioversion for this patient, which of the following would you choose in the ACUTE setting?

- Transesophageal echocardiogram with initiation of unfractionated heparin or low molecular weight heparin
- Unfractionated heparin or low molecular weight heparin
- Transesophageal echocardiogram without initiation of heparin products
- Warfarin only
- None of these
- Other ____________________
- Dabigatran

6. If you were to proceed to ELECTRICAL cardioversion for this patient, which of the following would you choose in the ACUTE setting?

- Transesophageal echocardiogram with initiation of unfractionated heparin or low molecular weight heparin
- Unfractionated heparin or low molecular weight heparin
- Transesophageal echocardiogram without initiation of heparin products
- Warfarin only
- None of these
- Other ____________________
- Dabigatran

SCENARIO 7: Please read the following scenario and answer the questions based on your general practice pattern. An 82-year-old woman with a history of PERMANENT atrial fibrillation on chronic anticoagulation with warfarin presents to the Emergency Department with acute onset palpitations and dyspnea for 8 hours. She is clear about the time of symptom onset. She is not taking any other cardiac medications. Vital signs: BP 135/70, HR 125, SpO2 96%RA.  EKG reveals atrial fibrillation. INR 2.5. Her CHADS2 score = 3 (Age, Hypertension, Diabetes). The patient has normal kidney function, no history of structural heart disease, a recent negative stress test, and no contraindications to anticoagulation.

1. In the acute setting, what would be the first step in your approach to this patient?

- Amiodarone
- Digoxin
- Diltiazem
- Direct Current Cardioversion
- Esmolol
- Ibutilide
- Metoprolol
- Procainamide
- Propafenone
- Verapamil
- Other ____________________

2. If your first choice was unsuccessful, what would be your next choice for management of this patient?

- Amiodarone
- Digoxin
- Diltiazem
- Direct Current Cardioversion
- Esmolol
- Ibutilide
- Metoprolol
- Procainamide
- Propafenone
- Verapamil
- Other ____________________

 Stand Alone Questions

1. Please indicate the optimal time frame for each of the following actions in the management of a NEW diagnosis of atrial fibrillation in the ACUTE setting.

|  | During index ED visit | Prior to hospital discharge | During outpatient follow-up | Not necessary |
| --- | --- | --- | --- | --- |
| Cardiac enzymes |  |  |  |  |
| Thyroid function |  |  |  |  |
| Echocardiogram |  |  |  |  |
| Stress test (treadmill/ chemical) |  |  |  |  |
| Cardiology consult |  |  |  |  |

Respondents who identified as Cardiology, Electrophysiology, or Emergency Medicine received question 2a. Respondents who identified as Hospital Medicine received question 2b.

2a. How likely are you to admit a patient with an ACUTE presentation of symptomatic atrial fibrillation/flutter in the following scenarios based on your general approach to this patient population?

|  | Not at all likely | Not very likely | Somewhat likely | Very likely |
| --- | --- | --- | --- | --- |
| New diagnosis atrial fibrillation |  |  |  |  |
| New diagnosis atrial flutter |  |  |  |  |
| Paroxysmal atrial fibrillation |  |  |  |  |
| Paroxysmal atrial flutter |  |  |  |  |
| Chronic atrial fibrillation |  |  |  |  |
| Chronic atrial flutter |  |  |  |  |

2b. How important is it to admit a patient with an ACUTE presentation of symptomatic atrial fibrillation/flutter in the following scenarios based on your general approach to this patient population?

|  | Not at all important | Not very important | Somewhat important | Very important |
| --- | --- | --- | --- | --- |
| New diagnosis atrial fibrillation |  |  |  |  |
| New diagnosis atrial flutter |  |  |  |  |
| Paroxysmal atrial fibrillation |  |  |  |  |
| Paroxysmal atrial flutter |  |  |  |  |
| Chronic atrial fibrillation |  |  |  |  |
| Chronic atrial flutter |  |  |  |  |

Respondents who identified as Hospital Medicine or Emergency Medicine received question 3a. Respondents who identified as Cardiology or Electrophysiology received question 3b.

3a. How likely are you to obtain a cardiology consult for the management of patients with ACUTE presentations of atrial fibrillation/ flutter in the following scenarios based on your general approach to this patient population?

|  | Not at all likely | Not very likely | Somewhat likely | Very likely |
| --- | --- | --- | --- | --- |
| New diagnosis atrial fibrillation |  |  |  |  |
| New diagnosis atrial flutter |  |  |  |  |
| Paroxysmal atrial fibrillation |  |  |  |  |
| Paroxysmal atrial flutter |  |  |  |  |
| Chronic atrial fibrillation |  |  |  |  |
| Chronic atrial flutter |  |  |  |  |

3b. How important is it to obtain a cardiology consult for the management of patients with ACUTE presentations of atrial fibrillation/ flutter in the following scenarios based on your general approach to this patient population?

|  | Not at all important | Not very important | Somewhat important | Very important |
| --- | --- | --- | --- | --- |
| New diagnosis atrial fibrillation |  |  |  |  |
| New diagnosis atrial flutter |  |  |  |  |
| Paroxysmal atrial fibrillation |  |  |  |  |
| Paroxysmal atrial flutter |  |  |  |  |
| Chronic atrial fibrillation |  |  |  |  |
| Chronic atrial flutter |  |  |  |  |

4. Please estimate how often you encounter patients with ACUTE presentations of atrial fibrillation in your practice.

- More than once a day
- About once a day
- Less than once a day up to once a week
- Less than once a week up to once a month
- Less than once a month
- Never

5. Which, if any, of the following guidelines for the management of acute presentations of atrial fibrillation have you EVER read or consulted?

- American College of Cardiology Foundation (ACC) / American Heart Association (AHA)
- European Society of Cardiology (ESC)
- American Academy of Family Physicians (AAFP)
- Heart Rhythm Society (HRS)
- None of these
- Other ____________________

If respondent selected any answer other than “None of these” to question #5 they received question #6. If they selected “None of these” to question #5, skip to Demographic questions.

6. How USEFUL do you think currently published practice guidelines available to you are in addressing management of ACUTE presentations of atrial fibrillation/ flutter?

- Not at all useful
- Not very useful
- Somewhat useful
- Very useful

Demographic questions.

1. Are you…

- Male
- Female
- Prefer not to answer

2. Which of the following ranges includes your age?

- 29 years or younger
- 30-39
- 40-49
- 50-59
- 60-69
- 70 or older
- Prefer not to answer

3. Please indicate the number of years you have been in practice after completion of your most advanced post-graduate training.

- 5 years or fewer
- 6-10 years
- 11-15 years
- 16-19 years
- 20 or more years

4. Which of the following best describes the practice setting where you spend the majority of your clinical time?

- Rural
- Suburban
- Urban

5. Which of the following best describes the practice setting where you spend the majority of your clinical time?

- Academic
- Community
- Mixed

6. Please select the state where you spend the majority of your clinical time.

- AL
- AK
- AR
- AZ
- CA
- CO
- CT
- DC
- DE
- FL
- GA
- HI
- IA
- ID
- IL
- IN
- KS
- KY
- LA
- MA
- MD
- ME
- MI
- MN
- MO
- MS
- MT
- NC
- ND
- NE
- NH
- NJ
- NM
- NV
- NY
- OH
- OK
- OR
- PA
- RI
- SC
- SD
- TN
- TX
- UT
- VA
- VT
- WA
- WI
- WV
- WY

7. What is the size of the hospital where you spend the majority of your clinical time?

- Fewer than 50 beds
- 50-150 beds
- 151-300 beds
- More than 300 beds
- Not sure
- I do not spend any clinical time in a hospital

8. What is the annual volume of the Emergency Department in the hospital where you spend the majority of your clinical time?

- Fewer than 25,000 patients
- 25,000-75,000 patients
- More than 75,000 patients
- Not sure
- I do not spend any clinical time in a hospital

If you are interested in being entered in a drawing for an iPad, please enter your email address below. ____________________________

Thank you for your time! Those are all the questions we have for you today.
